# Long-Term Nitrogen Removal Performance and Microbial Analysis in a SNAD-Based MBBR at Room Temperature

**DOI:** 10.3390/molecules31132325

**Published:** 2026-07-02

**Authors:** Xuejiao Yin

**Affiliations:** School of Architecture and Engineering, Chongqing Industry Polytechnic University, Chongqing 401120, China; yinxuejiao1012@126.com or yinxj@cqipu.edu.cn

**Keywords:** domestic sewage, *Candidatus Brocadia*, microbial community, C/N ratio, mainstream anammox

## Abstract

In recent years, the simultaneous partial nitrification, anammox, and denitrification (SNAD) process has attracted considerable attention due to its advantages such as low energy consumption and low sludge production. This study investigated the long-term performance and microbial mechanisms of a single-stage moving bed biofilm reactor (MBBR) employing the SNAD process for treating real domestic sewage at room temperature. A pre-carbon adsorption unit (reactor A) reduced the influent C/N ratio from 5.68:1 to 3.13:1, enabling efficient nitrogen removal in reactor B. Results demonstrated that dissolved oxygen (DO) and C/N ratio critically influenced system performance. At DO ~0.3 mg/L, stable SNAD operation achieved 65% total nitrogen (TN) removal, with synergistic contributions from partial nitrification, anammox, and heterotrophic denitrification. Elevated C/N ratios (4.71:1) reduced TN removal by ~30%, linked to decreased abundances of anammox bacteria. Microbial analysis revealed *Candidatus Brocadia* as key anammox bacteria, *Nitrospirae* as dominant ammonia-oxidizing bacteria and *Denitratisoma* as main denitrifying bacteria to drive nitrogen conversion. This study confirms the feasibility of SNAD-MBBR for real domestic sewage under ambient conditions, highlighting optimal DO and C/N control for microbial synergy and process stability.

## 1. Introduction

The denitrification technology based on anammox was considered as a revolutionary denitrification technology and has garnered widespread attention both domestically and internationally [1]. The sewage treatment process based on anammox does not require the consumption of organic carbon sources, making it a preferred option for treating low carbon-to-nitrogen ratio sewage and one of the most promising sewage treatment technologies currently available [2]. However, the current autotrophic anaerobic ammonium oxidation denitrification system has certain limitations. Firstly, for every 1 mol of ammonia nitrogen consumed, 0.11 mol of nitrate nitrogen was produced. This means that the maximum nitrogen removal efficiency in the PN/A system was 89%, and the remaining 11% of nitrogen will remain in the form of nitrate nitrogen in the wastewater [3,4]. In addition, organic matter was present in most nitrogen-containing wastewater and was difficult to completely remove.

In recent years, researchers have intensively investigated the application of. partial nitrification/anaerobic ammonium oxidation/denitrification (SNAD) denitrification process under low C/N organic nutrient conditions in wastewater treatment [5,6]. The SNAD process offers a sophisticated solution for nitrogen removal by synergistically integrating three microbial metabolic pathways within a single reactor [7]. Initially, ammonia oxidizing bacteria (AOB) partially convert ammonia to nitrite under oxygen-limited conditions. Subsequently, anammox bacteria utilize this nitrite to oxidize ammonia directly into dinitrogen gas, a reaction that provides the necessary electron acceptor for the third step. Finally, heterotrophic denitrifying bacteria (DNB) reduce this nitrate to nitrogen gas while oxidizing residual organic carbon [8]. This tripartite synergy allows SNAD to overcome the thermodynamic ceiling of conventional PN/A systems, where nitrate production theoretically caps TN removal at 89% [9]. By coupling anammox with endogenous or co-metabolic denitrification, the SNAD process achieves a near-zero nitrate yield, effectively pushing TN removal beyond 90% and presenting a highly efficient, energy-saving alternative for treating real domestic wastewater characterized by low C/N ratios [10,11].

Currently, the commonly used reactors for starting the SNAD process include the sequencing batch reactor (SBR), the membrane bioreactor (MBR), the sequencing batch biofilm reactor (SBBR) and the moving bed biofilm reactor (MBBR) [12,13,14]. The significant advantages of the SBR lie in its simplicity of operation and ease of parameter control. In addition, some important physiological parameters, such as biomass increment, maximum ammonia nitrogen consumption rate, maximum growth rate, and so on, are easier to monitor. However, the limitation of the SBR is that biomass is prone to loss [15]. The filter membrane in the MBR reactor has a retention effect on biomass, resulting in less biomass loss, which provides certain advantages for cultivating anaerobic ammonia-oxidizing bacteria with a longer doubling time. SBBR is a hybrid SBR process that involves suspended activated sludge and biofilm attached to freely flowing carriers. The main advantages of SBBR are its effective nitrogen removal under high loading rates, high biomass concentration achievable with short hydraulic retention time (HRT), high nitrite accumulation efficiency, and its suitability for low-temperature operation [14]. The MBBR have all the advantages of SBBR, using biofilm carriers with high surface area for bioretention was proven to be an economically feasible denitrification configuration. Compared with traditional activated sludge reactors, MBBR required a smaller reactor volume [16].

Previous studies on SNAD were mainly conducted at temperatures of 30–36 °C (the most suitable temperature for the growth of anammox bacteria), and the treated wastewater was mostly artificial wastewater containing nitrite and nitrate. There was relatively little research on the SNAD process for treating actual domestic sewage. The actual domestic sewage did not contain nitrite and nitrate, and the temperature was also below 30–36 °C. The treatment of actual domestic sewage by anaerobic ammonia oxidation bacteria was inevitably different from the treatment of artificial sewage under medium temperature conditions. Therefore, it was necessary to study the effectiveness and related mechanisms of SNAD process in treating actual domestic sewage.

This study aimed to remove some COD and reduce the C/N ratio in actual domestic wastewater through a pre carbon adsorption device at room temperature. The actual domestic sewage with reduced C/N ratio was used to start the SNAD system in a single-stage MBBR, and the effects of DO, aeration mode, C/N ratio and other factors on the denitrification efficiency of the SNAD system were explored. The microbial mechanism, including organic nutrient anaerobic ammonia oxidizing bacteria, in the system was analyzed by quantitative PCR and high-throughput sequencing. This study was distinguished from prior SNAD investigations in the following two ways: (i) it was operated at room temperature on real domestic sewage containing native COD and negligible nitrite instead of artificial synthetic sewage, directly addressing the mainstream application gap; (ii) it quantified the C/N–DO–aeration multi-factor response matrix alongside functional gene dynamics, providing engineering parameters for carbon-neutral WWTP retrofits seeking deep TN removal without external carbon dosing. The findings could provide mechanistic insight and engineering-scale parameters for deploying SNAD-MBBR in practical municipal wastewater treatment plants, particularly those seeking energy-efficient deep denitrification without external carbon addition.

## 2. Results and Discussions

### 2.1. COD and Nitrogen Removal Performance

#### 2.1.1. Reactor A Treatment Effect

Compared to urban sewage, student dormitory building sewage has the following characteristics: (1) it has poor comprehensiveness and a relatively single source, mainly consisting of bathing water and toilet flushing wastewater; (2) the amount and quality of sewage vary significantly over time. In addition, the seasonal variation in domestic sewage in universities was also quite obvious. The sewage volume in summer was significantly higher than that in winter, and the sewage volume in winter and summer vacation was significantly lower than usual. The concentration of pollutants was significantly higher from 1:00 am to 6:00 am compared to other time periods, with the lowest concentration at 22:00 and COD from Monday to Friday. The concentration of pollutants such as NH_4_^+^ was higher than on weekends. Overall, the fluctuation of sewage quality in student dormitory buildings can have a certain impact on the stable operation of domestic sewage treatment processes.

**Figure 1:**
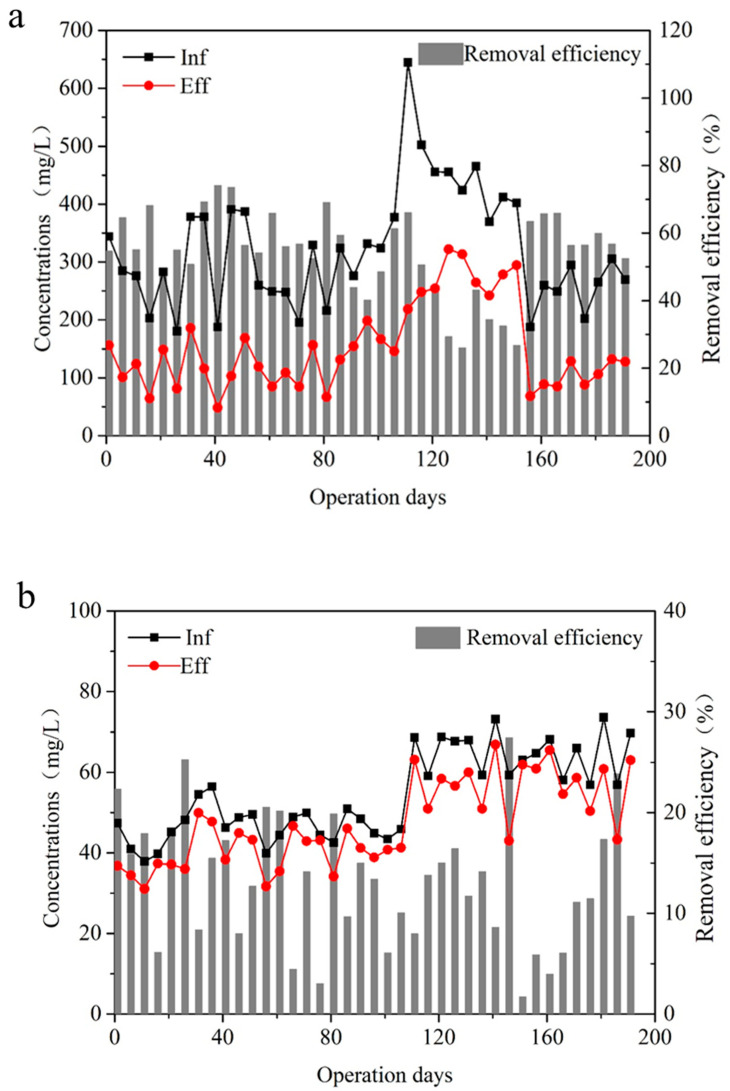
Concentrations of COD (**a**) and NH_4_^+^ (**b**) in the influent and effluent of reactor A.

The primary purpose of setting up reactor A was to remove COD, thereby reducing C/N. The specific monitoring results and removal rate analysis of COD content in the inlet and outlet water were shown in Figure 1a. In this study, within 0–106 days after the start of reactor A, the level of influent COD (dissolved state) was maintained at around 292.04 ± 69.14 mg/L, and the effluent COD was maintained at around 123.43 ± 41.45 mg/L. The COD removal rate was approximately 58.06% ± 9.34%. Within 111–151 days after startup, due to the high frequency of blockage in the sewage pipeline, the water inlet will be adjusted to open the sewage pipeline switch at regular intervals for sewage storage. This adjustment resulted in a longer retention time of sewage in the water storage tank and an increase in COD content in the influent. During this period, the COD content in the influent was around 459.06 ± 79.98 mg/L, and the COD in the effluent was around 270.69 ± 34.30 mg/L. The COD removal rate decreased to 39.23% ± 13.17%. The increase in COD content in the influent may be due to the longer residence time of the sewage, which allows microorganisms sufficient time to decompose solid organic matter in the sewage into dissolved organic matter. In addition, during this period, due to the prolonged residence time of sewage, anaerobic reactions such as acetic acid production, methane production, and H_2_S production may occur, leading to an increase in the content of toxic substances in the sewage and seriously affecting the sludge performance of reactor A. During this period, the sludge in reactor A was loose, with poor settling performance and a grayish white color. Thus, on the 152nd day, the sludge was retrieved from the sewage treatment plant and replaced with the original sludge. In addition, starting from day 152, the sewage pipeline switch will be adjusted back to the open state at any time. After adjustment, the sewage retention time will be shortened to avoid the problem of increased COD content in the influent of reactor A and deterioration of sludge performance. During days 156–191, the influent COD content remained at 254.34 ± 41.16 mg/L, the effluent COD content remained at around 103.18 ± 24.13 mg/L, and the COD removal rate increased to 59.66% ± 4.95%. Throughout the entire operation period, the influent COD content was approximately 322.85 ± 101.15 mg/L, and the effluent COD content remained at around 153.26 ± 74.92 mg/L. The COD removal rate was approximately 54.04% ± 12.53%.

During the operation of reactor A, the concentration and removal rate of ammonia nitrogen in the inlet and outlet water were shown in Figure 1b. The consumption of ammonia nitrogen was relatively stable. The average content of ammonia nitrogen in the inlet water was 54.37 ± 10.55 mg/L, and the average content of ammonia nitrogen in the outlet water was 47.39 ± 10.49 mg/L. The average removal rate of ammonia nitrogen was 13.08%. In addition, the average content of total inorganic nitrogen in the influent was about 56.84 mg/L, while the average content of total inorganic nitrogen in the effluent was about 48.74 mg/L. From the above results, it could be calculated that the average C/N (COD/TIN) level in the influent was 5.68:1, and the average C/N level in the effluent was 3.13:1, indicating a significant decrease in C/N.

#### 2.1.2. SNAD System Processing Effect

This study successfully initiated the SNAD process in MBBR B. Throughout the entire operation phase, the performance of reactor B was as follows:

During the 11th to 31st day (first stage), the average content of ammonia nitrogen in the influent of reactor B was 38.29 ± 6.98 mg/L, and the ammonia nitrogen content in the effluent was 0.45 ± 0.49 mg/L. The ammonia nitrogen removal rate was 98.91% ± 1.02%. The content of nitrite nitrogen in the inlet water was 0.21 ± 0.23 mg/L, and the content of nitrite nitrogen in the outlet water is 2.98 ± 1.98 mg/L. There was a certain accumulation of nitrite nitrogen in the outlet water, but the accumulation amount was relatively small. The nitrate nitrogen content in the influent was 1.61 ± 0.62 mg/L, and the nitrate nitrogen content in the effluent is 37.25 ± 6.31 mg/L. There was a significant accumulation of nitrate nitrogen before and after the reaction. The above monitoring results indicated that during this period, most of the ammonia nitrogen in reactor B was converted into nitrite nitrogen and nitrate nitrogen, and the main reaction occurring in the reactor was nitrification. It is speculated that the high dissolved oxygen content in the reactor may be due to continuous aeration. After testing, the dissolved oxygen content in reactor B was maintained at a level of 3.74 ± 0.26 mg/L.

On days 31–106 (second stage), the aeration mode was adjusted to intermittent aeration, and the dissolved oxygen level in the reactor was adjusted to 0.33 ± 0.20 mg/L. Considering that after 20 days of continuous aerobic cultivation, there may be significant growth and accumulation of nitrifying bacteria in B activated sludge, the activated sludge was re-inoculated from the sewage treatment plant at 31 days. After restarting, the average ammonia nitrogen content in the inlet water of reactor B during this time period was 41.09 ± 4.70 mg/L, and the ammonia nitrogen content in the outlet water gradually decreased from the initial 41.89 mg/L to a minimum of about 8.7 mg/L. The removal rate of ammonia nitrogen gradually increased from 12% to a maximum of 74%. During this period, no significant accumulation of nitrite nitrogen or nitrate nitrogen was found in the effluent. The average total nitrogen content in the influent was 42.42 ± 4.23 mg/L, and the total nitrogen removal rate increased from 10.03% at the beginning to a maximum removal rate of 67.30%. During this stage, the average COD content in the influent was 123.60 ± 42.01 mg/L, and the average COD content in the effluent was 68.04 ± 21.35 mg/L. The average removal rate of COD was 42.32%. The average C/N ratio in the inflow was 2.93:1. From day 66 to day 106, the removal rates of ammonia nitrogen and total nitrogen remained relatively stable, with ammonia nitrogen removal rate maintained at 65% ± 5.58% and total nitrogen removal rate maintained at 60.72% ± 5.04%. In addition, around the 96th day of start-up, significant red bacteria were observed in the B reactor, indicating that anaerobic ammonia oxidizing bacteria were significantly proliferating and accumulating during this period, and played an important role in nitrogen removal.

During the 111–151 days of start-up (third stage), there was a significant increase in COD in the influent. The average content of ammonia nitrogen in the influent was 56.88 ± 7.41 mg/L, and the average content of ammonia nitrogen in the effluent was 40.87 ± 5.17 mg/L. The average removal rate of ammonia nitrogen was 28% ± 3.97%. The average total nitrogen content in the influent was 58.48 ± 7.21 mg/L, and the average total nitrogen content in the effluent is 42.34 ± 5.02 mg/L. The average total nitrogen removal rate was 27.44% ± 4%. No significant accumulation of nitrite nitrogen and nitrate nitrogen was found in the effluent. During this stage, the average COD content in the influent was 270.69 ± 34.30 mg/L, and the average COD content in the effluent was 80.1 ± 23.09 mg/L. The average removal rate of COD was about 70.47%. The average C/N ratio in the inflow was 4.71:1. Compared to the previous stage, the removal rates of ammonia nitrogen and total nitrogen have significantly decreased. It can be seen that during this period, the removal of ammonia nitrogen and total nitrogen was affected to some extent due to the significant increase in COD in the influent. The C/N sensitivity demonstrated in Phases II–III–IV aligns well with literature findings. Chen et al. reported that anammox biofilm deteriorated when C/N exceeded 2.0, with specific anammox activity decreasing by 85% at C/N = 3.0 compared to C/N = 0 [17]. Similarly, Liu et al. observed TN removal in a SNAD–SBR decreasing from 92.8% at C/N = 2.0 to 76.8% at C/N = 3.5, with anammox contribution dropping from 63.1% to 48.1% [6]. The ~30% TN removal decline when C/N rose from 2.9:1 to 4.7:1 in this study, accompanied by a 33% reduction in *Candidatus Brocadia* abundance, is consistent with these reports. The metabolic plasticity of *Candidatus Brocadia* under mixotrophic conditions—where acetate can stimulate the DNRA pathway and cross-feeding with heterotrophs [18]—may explain why AnAOB persisted albeit at reduced abundance, rather than being fully washed out during the high-C/N Phase III. The rapid recovery upon C/N returning to 1.8:1 (TN removal rebounding by ~33%) further confirms the resilience of the MBBR biofilm architecture, which retains slow-growing AnAOB more effectively than suspended-sludge systems.

The COD level in the influent returned to normal on days 156–191 (fourth stage). The average content of ammonia nitrogen in the influent was 57.14 ± 7.34 mg/L, and the removal rate of ammonia nitrogen has increased from around 32.00% to a maximum of around 68.01%. The average total nitrogen content in the influent was 58.06 ± 7.37 mg/L, and the total nitrogen removal rate increased from 31.22% to a maximum of 65.11%. The average content of COD in the influent was 103.18 ± 24.12 mg/L, and the average content of COD in the effluent was 42.26 ± 12.81 mg/L, the average removal rate of COD was about 58.22%. The average C/N ratio in the inflow was 1.82:1.

**Figure 2:**
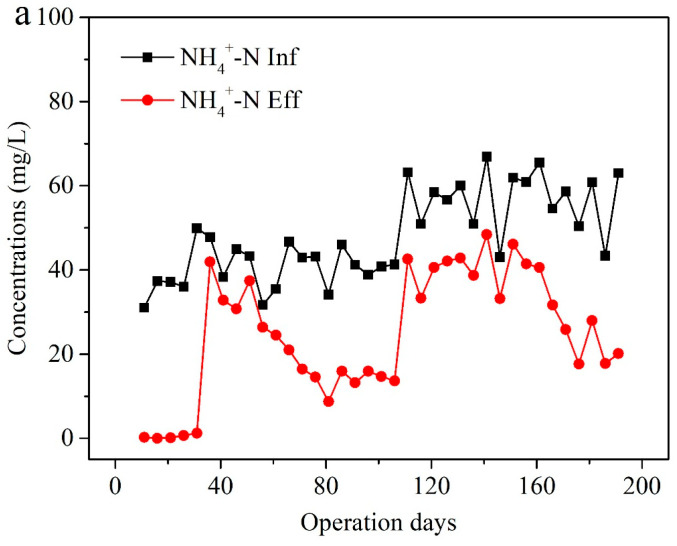

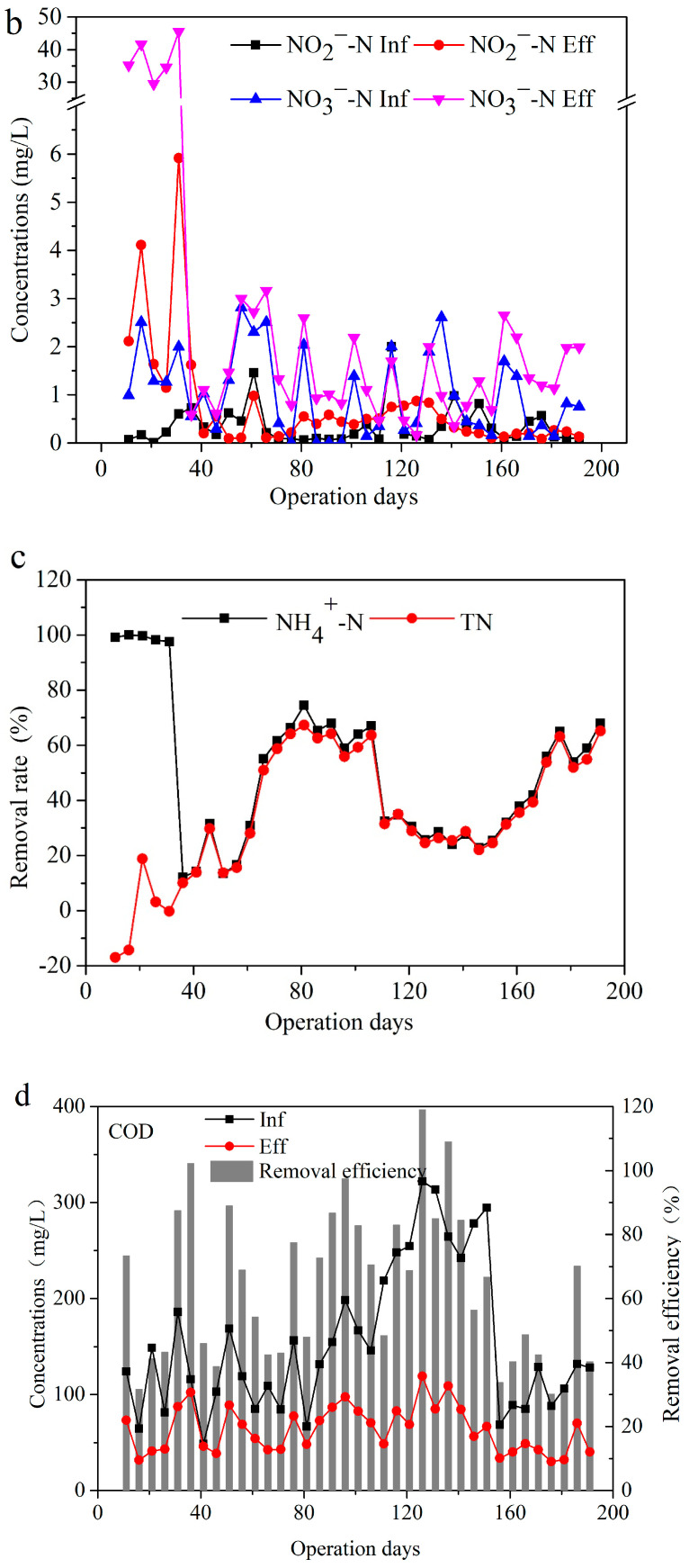
Nutrient removal in reactor B. (**a**): concentrations of ammonium in the influent and effluent; (**b**): concentrations of nitrite and nitrate in the influent and effluent; (**c**): removal efficiency of ammonium and total inorganic nitrogen; (**d**): COD concentration in the influent and effluent and COD removal efficiency.

The monitoring results of inorganic nitrogen compounds and COD in the inlet and outlet of reactor B, as well as the removal rates of ammonia nitrogen and total nitrogen, are shown in Figure 2.

The COD removal rate of reactor B during the entire operation period was 54.02% ± 16.76%. From the above results, it can be seen that B mainly undergoes nitrification reaction in the first stage, where almost all ammonia nitrogen is converted into nitrate nitrogen, and the total nitrogen removal effect is not ideal. In the second stage, the removal rate of total inorganic nitrogen in B was the highest at 67.30%, and at the end of the second stage (66–106 days), the removal rate of total inorganic nitrogen remained at 60.72% ± 5.04%. The average total nitrogen removal rate in the third stage is 27.44% ± 4%. The highest total nitrogen removal rate in the fourth stage reached 65.11%, and the removal of total nitrogen was relatively stable at the end of the fourth stage. It can be seen that stable denitrification systems have been formed in reactor B in both the second and fourth stages. In this stable denitrification system, due to the fact that the influent mainly contains ammonia nitrogen, nitrification/denitrification was an essential step. In addition, the emergence of the “red bacteria” phenomenon in the system indicates that anaerobic ammonia-oxidizing bacteria have proliferated and played a role in denitrification in the system.

The nitrogen removal performance observed in this study was comparable to, yet distinct from, previously reported SNAD systems treating various wastewaters. Ding et al. achieved 86.1% TN removal treating real domestic sewage with a C/N of 3.0–3.5 using suspended activated sludge SNAD–SBR, without COD pretreatment, and reported only 1.02 mg/L nitrate in the effluent [19]. However, the SNAD-SBR system reported by Ding et al. was operated at the temperature of about 30 °C by a heater, which is difficult to achieve in real wastewater treatment. Wang et al. reported an optimal TN removal of 86.4% at C/N = 1.0 in a SNAD–MBR fed with synthetic wastewater, where anammox contributed 64.7% of total nitrogen removal [20]. In contrast, Zhou et al. attained 79.7% TN removal in a pilot-scale SNAD–MBBR treating swine digester liquor at 35 °C and DO = 0.07–0.30 mg/L, with much higher nitrogen loading (1.09 kg TN/m^3^·d). The moderate TN removal (65%) achieved here under room-temperature, real domestic sewage, and pre-adsorbed C/N ≈ 3.1 conditions reflects the inherent challenge of mainstream anammox application, where lower temperature and influent fluctuation constrain AnAOB activity [21].

Compared to other reactor configurations, the MBBR carrier system (60% filling ratio) proved advantageous for maintaining SNAD consortia under fluctuating C/N. While Cao et al. demonstrated successful full-scale SNAD with NRR = 0.9 kg/(m^3^·d) at high ammonia (500 mg/L NH_4_^+^–N) [5], and Wang et al. reported SNAD–MBBR for coal-gasification wastewater achieving 90.7% TN removal at 30–33 °C, this study is among the few confirming SNAD feasibility in single-stage MBBR treating real domestic sewage at ambient temperature with a simple pre-carbon adsorption step [20]. The integration of pre-adsorption (reactor A) to actively lower C/N from 5.68:1 to 3.13:1 represents a pragmatic strategy for mainstream application, bridging the gap between high-C/N reality of municipal sewage and the low-C/N preference of anammox.

**Figure 3:**
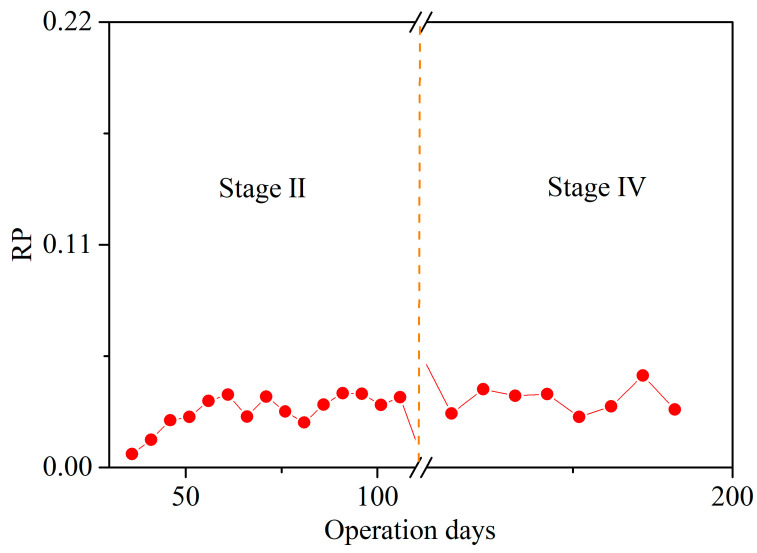
RP (increase in nitrate/consumption of nitrite + ammonium) value in reactor B.

The ratio of nitrate nitrogen increased to nitrite nitrogen consumption + ammonia nitrogen consumption (RP) in the second and fourth stages of reactor B was shown in Figure 3. The theoretical value of RP when only autotrophic anammox reaction occurs in the system was 0.11, which means that the maximum removal ratio of total nitrogen when only autotrophic anaerobic ammonia oxidation reaction occurs in the system was 89%. In the second and fourth stages, the average RP value inside reactor B was around 0.022, much lower than 0.11, breaking through the limitations of autotrophic anaerobic ammonia oxidation denitrification.

### 2.2. Microbial Monitoring Results in SNAD

#### 2.2.1. Absolute Quantitative PCR (qPCR)

**Figure 4:**
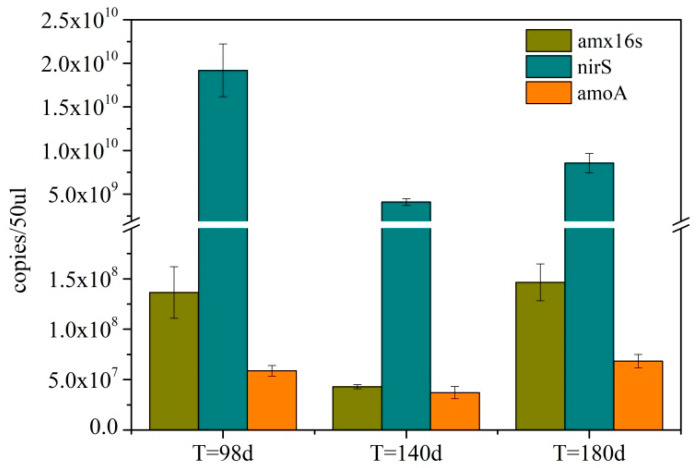
Abundances of nitrogen metabolism related functional genes in reactor B at different operation time. amx16s: anammox 16S rRNA gene; nirS: nitrite reductase functional gene; amoA: ammonia oxidase functional gene.

For reactor B, due to the lack of a good denitrification system in the first stage, further analysis of the system in the first stage was not conducted during operation. During the second stage (98 days), third stage (140 days), and fourth stage (180 days) of operation, sludge samples were taken from the reactor to perform absolute quantitative PCR detection of the abundance of ammonia oxidation functional gene amoA, denitrification functional gene nirS, and anaerobic ammonia oxidation 16S rRNA gene in the samples. The anaerobic ammonia oxidation 16S rRNA gene is a marker gene for anaerobic ammonia oxidation bacteria (AnAOB), and its abundance can represent the abundance of anaerobic ammonia oxidation bacteria in the system. The amoA functional gene is a marker gene for ammonia-oxidizing bacteria (AOB), and its abundance can represent the abundance of AOB in the system. The genomes of various anaerobic ammonia-oxidizing bacteria contain nirS functional genes, and nirS is also an important nitrogen metabolism functional gene in the genome of denitrifying bacteria (DNB). Some other symbiotic bacteria, such as *Chlorobi* and *Chloroflex*, can also contain nirS functional genes in their genomes, which play a role in partial denitrification [22]. Therefore, the abundance of nirS functional genes is the total number of bacteria in the system that can perform nitrite reduction processes. The results of absolute quantitative PCR detection were shown in Figure 4. In the second stage (98 days), the abundance of anaerobic ammonia oxidation 16S rRNA, denitrification functional genes, and ammonia oxidation functional genes were 1.37 × 10^8^, 1.92 × 10^10^, and 5.88 × 10^7^ copies/50 μL DNA samples, respectively. In the third stage (140 days), the detection results showed that the abundance of anaerobic ammonia oxidation 16S rRNA, denitrification functional genes, and ammonia oxidation functional genes were 4.30 × 10^7^, 4.10 × 10^9^, and 3.17 × 10^7^ copies/50 μL DNA samples, respectively. Compared to the previous stage, the abundance of 16S rRNA gene, nirS gene, and amoA gene has significantly decreased in this stage, with a decrease rate of 71%, 52%, and 46%, respectively. This may be due to the increase in COD content in the third-stage matrix inhibiting the activity of AOB and AnAOB, as well as the decrease in DNB abundance due to substrate deficiency. In the fourth stage (140 days), the COD level in the reactor inlet water returned to normal. The test results showed that the abundance of anaerobic ammonia oxidation 16S rRNA, denitrification functional genes, and ammonia oxidation functional genes in this stage were 1.46 × 10^8^, 8.56 × 10^9^, and 6.83 × 10^7^ copies/50 μL DNA sample, respectively. The abundance of 16S rRNA gene and amoA gene in the fourth stage system has increased compared to the third stage. In addition, compared to the second stage, the abundance of nirS gene decreased slightly in the fourth stage, while the abundance of 16S rRNA gene and amoA gene increased slightly. This may be due to the lower COD content in the matrix in the fourth stage compared to the second stage. From the quantitative PCR results, it can be seen that there must be AnAOB and AOB present in the system, and the abundance of AnAOB was higher than that of AOB. Additionally, there may be DNBs present in the system. The specific microbial composition in the system needs further high-throughput sequencing verification.

#### 2.2.2. Microbial Community

**Figure 5:**
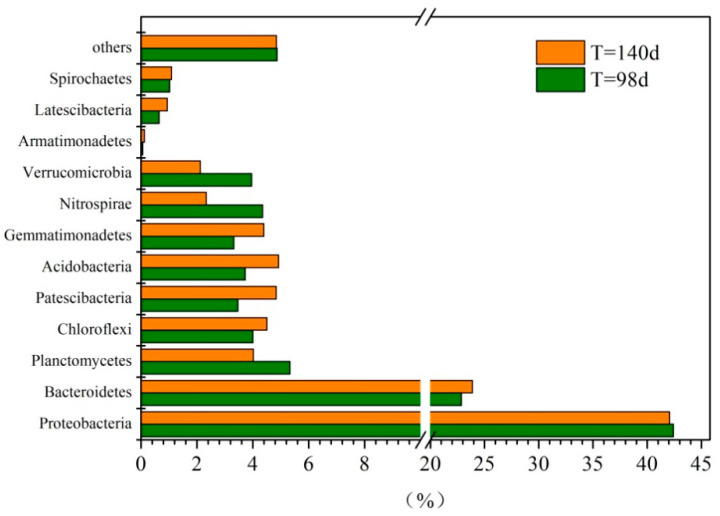
Bacterial abundances in reactor B on genus level.

For reactor B, due to the good and stable denitrification performance at the end of the second stage, while in the third stage, the denitrification performance deteriorates due to the increase in COD level in the influent, and the denitrification performance in the fourth stage was relatively similar. Therefore, representative late stage and late stage of the second and third phases were selected for high-throughput sequencing. In the second stage (98 days) and third stage (140 days) of operation, sludge samples were taken from the reactor for high-throughput sequencing to obtain information on the composition of microorganisms in the system. The composition of microorganisms in the system obtained through high-throughput sequencing and analysis was shown in Figure 5. The high-throughput sequencing results indicated that at the phylum level, the microorganisms in B mainly consist of *Proteobacteria, Bacteroidetes*, *Planctomycetes*, *Chloroflexi*, *Patescibacteria, Acidobacteria*, *Gemmatimonadetes*, *Nitrospirae*, *Verrucomicrobia*, *Armatimonadetes*, *Latescibacteria* and *Spirochaetes*; these microorganisms account for over 95% of the total microbial abundance. Among nitrogen metabolism related bacteria, AnAOB belongs to Planctomycetes, AOB mainly belongs to *Nitrospirae* and *Proteobacteria*, DNB mainly belongs to *Proteobacteria* and *Bacteroidetes*, and *Chloroflexi* was a common symbiotic bacterium in anaerobic ammonia oxidation denitrification systems. Some *Chloroflexi* bacteria can complete partial denitrification processes [23]. At the end of the second stage, the proportion of various microorganisms was 42.4% *Proteobacteria*, *Bacteroidetes* 22.86%, *Planctomycetes* 5.33%, *Chloroflexi* 4.00%, *Patescibacteria* 3.46%, *Acidobacteria* 3.73%, *Gemmatimonadetes* 3.32%, *Nitrospirae* 4.35%, *Verrucomicrobia* 3.96%, *Armatimonadetes* 0.06%, *Latescibacteria* 0.65% and *Spirochaetes* 1.02%. At the end of the third stage, the proportion of various microorganisms was 42.06% *Proteobacteria*, *Bacteroidetes* 23.86%, *Planctomycetes* 4.02%, *Chloroflexi* 4.5%, *Patescibacteria* 4.83%, *Acidobacteria* 4.92%, *Gemmatimonadetes* 4.39%, *Nitrospirae* 2.33%, *Verrucomicrobia* 2.12%, *Armatimonadetes* 0.12%, *Latescibacteria* 0.94% and *Spirochaetes* 1.09%. Compared to the end of the second stage, the content of *Planctomycetes* decreased by 1.31% in the third stage. The content of *Nitrospirae* decreased by 2.02%, while the relative abundance of *Proteobacteria* and *Bacteroidetes* did not show significant changes. The horizontal microbial categories and contents related to nitrogen metabolism in the system are shown in Table 1. According to Table 1, at the end of the second and third stages of B operation, the total relative abundance of DNB were 16.851% and 16.844%, respectively, and there was no significant change in the total abundance of denitrifying bacteria. The DNB species with a relative abundance exceeding 1% in the system were *Rhodobacteraceae*, *Zoogloea, Thauera*, *Denitratisoma*, *Dechlomonas*, and *Bdellovibrio*, with the most notable being *Denitratisoma*. According to reports, *Denitratisoma* was a type of aerobic denitrifying bacteria that can directly oxidize nitrite into nitrogen gas. Compared to the second stage, there was no significant change in the relative abundance of *Denitratisoma* in the third stage; the abundance of *Rhodobacteraceae* increased significantly, from 1.19% to 3.5%. The abundance of *Zoogloea* significantly decreased from 2.66% to 0.12%. The abundance of *Thauera* had been significantly increased from 1.79% to 3.32%. The relative abundance of *Dechloromonas* has significantly decreased, from 2.91% to 1.37%. The relative abundance of *Bdellovibrio* had been slightly decreased, from 1.15% to 0.72%. At the end of the second and third stages of reactor B operation, the total relative abundance of AOB and NOB was 4.51% and 2.56%, respectively. Compared to the second stage, the content of AOB and NOB in the third stage decreased significantly. This may be due to the increase in COD in the influent, which consumes some dissolved oxygen, resulting in a decrease in dissolved oxygen content and affecting the activity of AOB. Additionally, AOB and NOB are autotrophic microorganisms, and studies had shown that an increase in COD can inhibit the activity of AOB [24,25]. The NOB types in reactor B were *Nitrosomonadaceae*, *Omnitrophicaeota*, *Nitrospira*; the most significant among them was *Nitrospira*. The AOB type in the system was *Nitrosomonas*, and the abundance of AOB was much lower than that of NOB. In the third stage, the relative abundance of *Nitrospira* decreased from 4.3% to 2.3%. The main type of AnAOB in B was the organic trophic anaerobic ammonia-oxidizing bacterium *Candidatus Brocadia*, which is consistent with the report of Li et al., and its relative abundance decreased from 4.76% to 3.2% in the third stage. The decrease in AnAOB abundance may be due to the increase in COD in the influent. Research had shown that C/N was a key factor affecting the activity of AnAOB. After the C/N ratio inflow increased from 2.93 ± 1.03:1 to 4.71 ± 0.92:1 in the third stage, anaerobic ammonia-oxidizing bacteria were significantly inhibited, with an abundance of only 67% of the second stage.

### 2.3. Nitrogen and Carbon Conversion Mechanism in SNAD System

**Figure 6:**
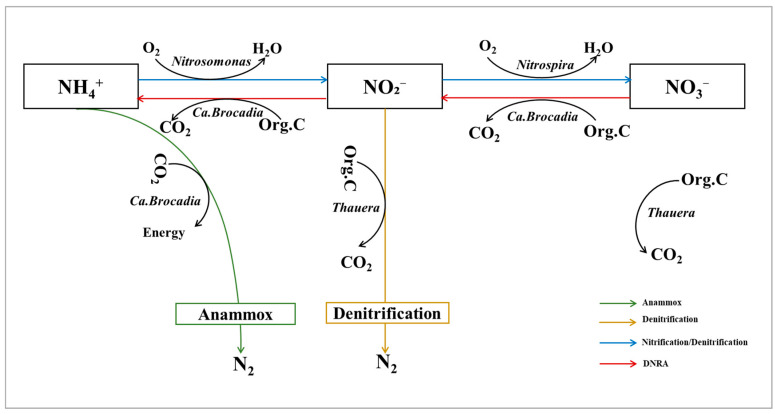
Synergistic transformation mechanism of C and N under microorganism regulation in reactor B.

SNAD system was formed in reactor B during the second and fourth stages. Partial nitrification, denitrification, and anaerobic ammonium oxidation combined to remove nitrogen and carbon from the substrate of B reactor. According to Section 2.2, the bacteria that complete ammonia oxidation were AOBs mainly composed of *Nitrosomonas*, while the bacteria that complete nitrification processwere NOBs mainly composed of *Nitrospira.* AOB and NOB consume dissolved oxygen in the system while converting ammonia nitrogen and nitrite. The bacteria that complete denitrification were DNBs represented by *Denitratshima* and *Thauera*. During the denitrification process, DNB uses organic carbon as energy to convert it into carbon dioxide. The AnAOB mainly composed of *Candidatus Brocadia fulgida* completes anaerobic ammonia oxidation. AnAOB fixes inorganic carbon while completing the anaerobic ammonia oxidation process, converting it into energy that can be used for its own purposes. Furthermore, *Candidatus Brocadia fulgida* exhibited organic properties and can utilize organic carbon in small amounts through the DNRA pathway in the presence of organic carbon [26]. In the DNRA pathway, nitrate was reduced to nitrite and further reduced to ammonia nitrogen. Based on the above results, the conversion mechanism of C and N in the SNAD system formed in reactor B was shown in Figure 6.

### 2.4. Future Perspectives

To further enhance TN removal, future work should focus on (i) developing advanced carriers to improve biofilm retention and AnAOB activity, (ii) fine tuning multi parameter interactions (DO, C/N, HRT, temperature) via data-driven models, and (iii) mitigating low temperature inhibition through NOB suppression or cold-adapted strain enrichment. For upscaling, pilot-scale trials are essential to validate performance under real-world hydraulic fluctuations and to assess the impacts of coexisting inorganic and organic micropollutants on microbial synergy. Long-term monitoring of functional gene dynamics and resilience strategies should be integrated. These efforts will bridge the gap from lab scale to mainstream municipal WWTPs, ensuring robust SNAD MBBR operation under variable influent quality and ambient conditions.

## 3. Materials and Methods

### 3.1. Reactor Design

**Figure 7:**
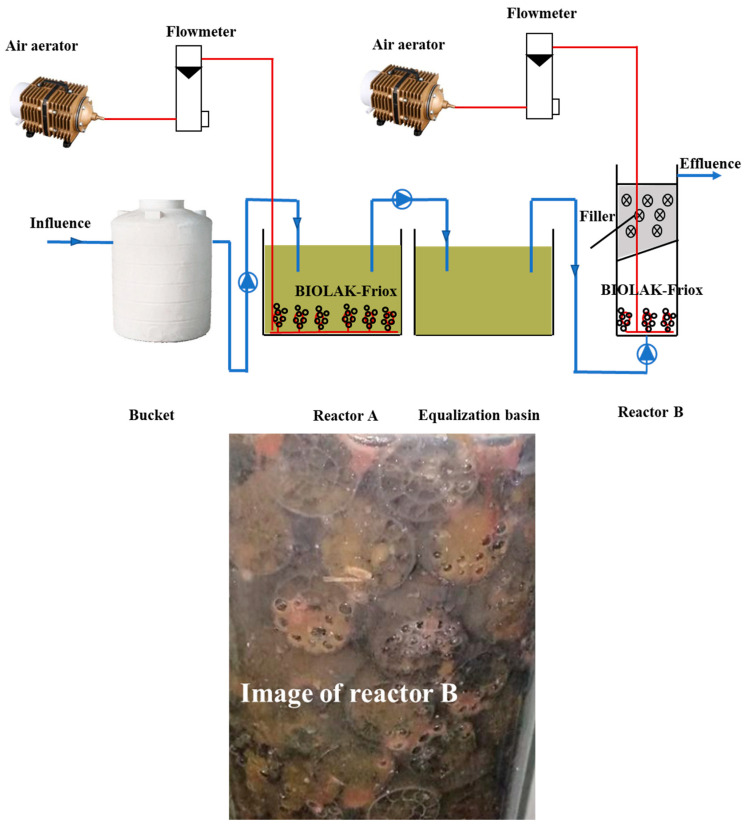
A-B sewage treatment process.

This experiment adopts the A-B sewage treatment process. The process flow chart was shown in Figure 7. The original sewage was from the student dormitory (29.5674° N, 106.4595° E). The raw sewage was pumped into reactor A by a peristaltic pump after passing through a 300 L storage tank (white plastic bucket). Reactor A was a pre-carbon capture device, where some COD was consumed and the C/N ratio was significantly reduced. Reactor A was an open-top cubic reactor constructed from transparent acrylic, with a total volume of 12 L. The effluent from reactor A was pumped into the regulating tank by a peristaltic pump, and the outlet was approximately 2/3 of the height of the reactor. The regulating pool played a role in regulating the water volume and supplying inlet water to reactor B. Reactor B was an open-top cylindrical moving bed biofilm reactor (MBBR) for denitrification, constructed from transparent acrylic, with an internal diameter of 150 mm, a height of 500 mm, and an effective volume of approximately 7.9 L. The reactor was filled with MBBR filler inside. In this study, in order to provide more biomass attachment carriers while ensuring sufficient aeration conditions, the MBBR packing ratio was appropriately increased to around 60%. The sewage was pumped by a peristaltic pump from the regulating tank to the bottom of reactor B, and the effluent method was overflow effluent.

### 3.2. Operation of the Reactor

The main purpose of setting up reactor A was to carry out carbon absorption and adsorption, remove some COD and insoluble nitrogen, and reduce the C/N level in the influent of reactor B. In this study, reactor A was operated using SBR method. Hydraulic retention time, aeration rate, and sludge settling time are key factors for the successful start-up of reactor A [27]. Excessive hydraulic retention time or aeration rate can lead to the accumulation of nitrifying bacteria in reactor A, resulting in the removal of COD while converting ammonia nitrogen into nitrate nitrogen, which was not conducive to the successful start-up of some nitrite/anaerobic ammonia oxidation systems. Short sedimentation time of sludge can lead to incomplete sedimentation of sludge, resulting in a large amount of sludge loss in reactor A. Excessive sedimentation time of sludge could lead to insufficient dissolved oxygen content, thereby affecting the removal of COD. In this study, the circulation period of reactor A was 60 min. Its operating mode was water inlet for 10 min, aeration (45 W air blower) for 30 min, sedimentation for 20 min, and effluent for 10 min.

Reactor B was initiated on day 11, after reactor A had reached stable operation. Prior to start-up, we first loaded the carriers to 60% of the reactor volume and then inoculated the system with activated sludge together with the effluent from reactor A. To promote sludge adhesion onto the carrier surface, we applied a 12 h static aeration (closed aeration) period, during which air was supplied continuously from the reactor bottom to facilitate initial biofilm formation on the carriers. The activated sludge deposited at the bottom of the reactor floats up and down under the disturbance of the air, constantly coming into contact with the packing material in the reactor. Eventually, some of the activated sludge successfully adheres to the packing material. Throughout the experimental period (April to November), the reactor temperature was maintained at 15–35 °C without additional heating or cooling. Aeration was carefully controlled, for that excessive air flow could lead to excessive shear force and make it difficult for sludge to adhere; if the air flow rate was too low, it will be difficult for the sludge to float up and come into full contact with the packing material. Finally, the air flow rate was controlled at around 1.5 L/min. After the end of the exposure, reactor B officially started to be operated. Each cycle lasted 6 h, consisting of a 20 min fill phase and a filling volume equivalent to 50% of the reactor volume. After the water inlet was completed, aeration (air) begins. During the first 11–31 days, the aeration time for reactor B was 5 h, the aeration flow rate was 0.5 L/min, and the sedimentation time was 40 min. After 31 days, in order to reduce the dissolved oxygen content in the reactor, the aeration measures were adjusted to aeration for 30 min, stopped aeration for 30 min, and the aeration flow rate was adjusted to 0.3 L/min.

### 3.3. Filler

The filler used in this experiment was a modified biological suspension filler (Dalian Yudu Environmental Company, Dalian, China, model WD-F25) made of high-density polyethylene, with a diameter and height of 25 mm and 10 mm respectively, and a relative density of 0.96–0.98 g/cm^3^. It had the advantages of large specific surface area, easy film formation, and good hydrophilicity, and had been widely used in various practical projects.

### 3.4. Sludge

The source of activated sludge used in this experiment was Chongqing Jiguanshi Wastewater Treatment Plant (Chongqing, China). The sludge from reactor A and reactor B was taken from the aerobic zone. After vaccination, the initial MLSS of reactor A was 4000 mg/L, and the initial MLSS of reactor B was about 2500 mg/L.

### 3.5. Chemical Analysis

A total of 50 mL supernatant samples were filtered before the analysis. The analyzed chemical index included ammonium, nitrite and nitrate. The filtration was carried out on filter membranes (Jinteng, Zhoushan, China) with a pore size of 0.45 μm. The ammonium (using the Nesslerization method (HJ 533-2009 [28], Chinese standard)), nitrate (using the N-(1-naphthyl)-ethylenediamine spectrophotometric method (GB/T 13580.7-1992 [29], Chinese National Standard)) and nitrite (using ultraviolet spectrophotometry (HJ/T 346-2007 [30], Chinese standard)) were analyzed three times in parallel by a colorimetric assay using UV-2550 ultraviolet spectrophotometer (Shimadzu, Nakagyo-ku, Japan). COD concentrations were determined using a spectrophotometer (HACH DR2800, Loveland, CO, USA) according to the rapid digestion spectrophotometric method (HJ/T 399-2007 [31]).

### 3.6. Microbial Analysis

#### 3.6.1. DNA Extraction

A total of 5 mL of sample was taken from the reactor, centrifuged at 8000 rpm for 5 min, and poured out the supernatant. E Z.N.A. ^®^ Soil DNA kit (Omega Bio tek, Norcross, GA, USA) was used to extract microbial genomic DNA from the sample according to the instructions.

#### 3.6.2. Fluorescence Quantitative PCR (qPCR)

In this experiment, in order to obtain the abundance of denitrifying bacteria, genomic DNA was extracted from 5 mL of the reactor sample, and qPCR was used to quantitatively analyze nitrogen conversion genes including anammox 16S rRNA (Amx 16S) gene, ammonia oxidation functional gene (amoA), and denitrification functional gene (nirS). The experimental process was as follows: QPCR was performed in a 15 μL reaction system containing 7.5 μL of SYBR green premix (China Genstar), 0.3 μL of forward and reverse primers, 0.3 μL of genomic DNA, and 6.6 μL of ddH_2_O. The primer information for qPCR was shown in Table 2.

In the application experiment of organic nutrient anaerobic ammonium oxidation bacteria in domestic sewage treatment, high-throughput sequencing was performed on sludge samples from reactor B in the second and third stages. During the sequencing process, samples with T = 98 d and T = 140 d produced 26,816 and 29,342 gene fragments, respectively, with a base number of 11,314,086 and 12,392,812. The length of the gene fragment was 358–515 bp and 335–524 bp.

The primers used for high-throughput sequencing were bacterial universal primers 338F (5′-ACTCCACGGGAGGCAGCAG-3′) and 806R (5′-GACTACHVGGGTWTCTAAT-3′). Usearch (http://www.drive5.com/usearch/ (accessed on 20 April 2026) Version 7.0) was used for OUT statistics; Uparse (http://www.drive5.com/uparse/ (accessed on 20 April 2026) Version 7.0.1090) was used for OUT clustering analysis. Use RDP classifier Bayesian algorithm to perform taxonomic analysis on OTU representative sequences with 97% similarity level, and calculate the community species composition of each sample. Compare the database to Silva (Release 132) (http://www.arb-silva.de).

#### 3.6.3. Statistical Analysis

The abundances of anammox 16S rRNA gene and two functional genes, namely nirS and amoA, were used in stepwise regression analyses with SPSS Statistics 20 (IBM, Armonk, NY, USA). A statistical One-Way ANOVA was also applied to analyze these results by SPSS Statistics 20 (IBM, USA)

## 4. Conclusions

This study demonstrated the feasibility of a SNAD–MBBR system for treating real domestic sewage at room temperature. Key findings were: (1) the MBBR with 60% carrier filling facilitated biofilm formation, achieving a stable TN removal efficiency of 65% at DO ~0.3 mg/L. (2) Heterotrophic denitrification effectively consumed nitrate byproducts (RP = 0.022), overcoming the intrinsic 89% TN removal limit of conventional anammox. (3) System performance was highly sensitive to operational parameters; optimal synergy occurred at DO ~0.3 mg/L, whereas high DO (~3.74 mg/L) favored nitrification. Crucially, increasing the C/N ratio from 2.9 to 4.7 suppressed *Candidatus Brocadia*(AnAOB) abundance (to 67% of baseline) and caused a ~30% drop in TN removal. (4) Microbial analysis revealed a robust consortium dominated by *Candidatus Brocadia*(AnAOB), *Nitrospira* (AOB), and *Denitratisoma* (DNB), confirming the functional stability of the SNAD ecosystem. This study provides a compact, energy-efficient solution for low-C/N wastewater treatment.

## Figures and Tables

**Table 1:** Nitrogen metabolism related bacteria and their abundances in reactor B.

Genus	98d (%)	140d (%)	Genus	98d (%)	140d (%)
DNB			DNB		
*Rhodanobacteraceae*	1.19	3.5	*Flavobacterium*	0.48	0.09
*Enterobacteriaceae*	0.01	0	*Dokdonella*	0.54	0.4
*Zoogloea*	2.66	0.12	*Devosia*	0.02	0.03
*Thiothrix*	0.03	0.02	*Denitratisoma*	4.13	4.48
*Thiobacillus*	0.07	0.28	*Defluviimonas*	0.04	0.08
*Thermomonas*	0.2	0.2	*Dechloromonas*	2.91	1.37
*Thauera*	1.79	3.32	*Cloacibacterium*	0.03	0.03
*Terrimonas*	0.5	0.79	*Arenimonas*	0.04	0.38
*Sphingobium*	0.07	0.03	*Bdellovibrio*	1.15	0.72
*Roseomonas*	0.005	0.04	Total	16.851	16.844
*Pseudoxanthomonas*	0.03	0.06	AOB and NOB		
*Pseudomonas*	0.13	0.11	*Nitrosomonadaceae*	0.03	0.05
*Pedobacter*	0.006	0.009	*Omnitrophicaeota*	0.05	0.11
*Opitutus*	0.12	0.05	*Nitrospira*	4.3	2.3
*Nakamurella*	0.01	0.02	*Nitrosomonas*	0.13	0.10
*Mesorhizobium*	0.06	0.04	Total	4.51	2.56
*Hydrogenophaga*	0.15	0.005	AnAOB		
*Haliangium*	0.48	0.67	*Candidatus Brocadia*	4.76	3.20

**Table 2:** Sequences, annealing temperatures and target genes of the primers used in this study.

Primer	Sequences (5′-3′)	Annealing Temperature (°C)	Target Gene	References
338F	ACTCCTACGGGAGGCAGCA	52	Anammox 16S rRNA	Du et al., 2019 [32]
820R	TTCGCAATGCCCGAAAGG			
1F	GGGGTTTCTACTGGTGGT	55	amoA	Han et al., 2020 [33]
2R	CCCCTCKGSAAAGCCTTCTTC			
cd3AF9	GTSAACGTSAAGGARACSGG	55	nirS	Yin et al., 2021 [34]
R3cd	GASTTCGGRTGSGTCTTGA			

## Data Availability

The data presented in this study are available on request from the corresponding author due to the large volume and unstructured nature of the experimental datasets.
